# Safety and performance of the Medtronic 3830 lead in His-bundle and Left bundle branch area pacing: A single-center experience

**DOI:** 10.1007/s10840-025-02070-3

**Published:** 2025-06-14

**Authors:** Alejandro Sanchez-Nadales, Abdullah Sarkar, Jose Sleiman, Andres Sanchez-Nadales, Mileydis Alonso, John Bibawy, Marcelo Helguera, Sergio Pinski, Jose Baez-Escudero

**Affiliations:** Robert and Suzanne Tomsich Department of Cardiology, Department of Cardiovascular Disease, Cleveland Clinic Florida, Weston Campus, Weston, FL 33324 USA

**Keywords:** SelectSecure 3830, Conduction system pacing, His bundle pacing, Left bundle branch area pacing

## Abstract

**Background:**

Conduction system pacing (CSP) using His Bundle Pacing (HBP) and Left Bundle Branch Area Pacing (LBAP) is an evolving alternative to traditional right ventricular pacing (RVP), promising better physiological outcomes. This study evaluates the safety, feasibility, and performance of HBP and LBAP with Medtronic SelectSecure 3830 leads.

**Methods:**

We conducted a single-center retrospective analysis of 490 patients undergoing HBP or LBAP. The study assessed implant success rates, pacing thresholds, device longevity, and complication rates over an average follow-up of 28 months for HBP and 14 months for LBAP.

**Results:**

The implantation success rate was 85% for HBP and 97.4% for LBAP. LBAP demonstrated lower and more stable pacing thresholds, with initial values of 0.8V at 0.5 ms rising slightly to 0.9V at 0.5 ms, and fewer device revisions compared to HBP, whose initial pacing threshold of 1.3V at 0.8ms increased to 1.68 V at 0.7ms. Complications were minimal and similar across both groups. The need for fewer device revisions and potential for prolonged device life highlighted LBAP as potentially more cost-effective. Cardiac function measured by LVEF remained stable across both groups.

**Conclusions:**

Both HBP and LBAP are safe and feasible with comparable safety profiles. LBAP may offer advantages in terms of stability, fewer revisions, and extended device longevity. The study underscores the need for further research into optimal lead positioning and long-term outcomes of CSP, particularly for LBAP.

**Graphical Abstract:**

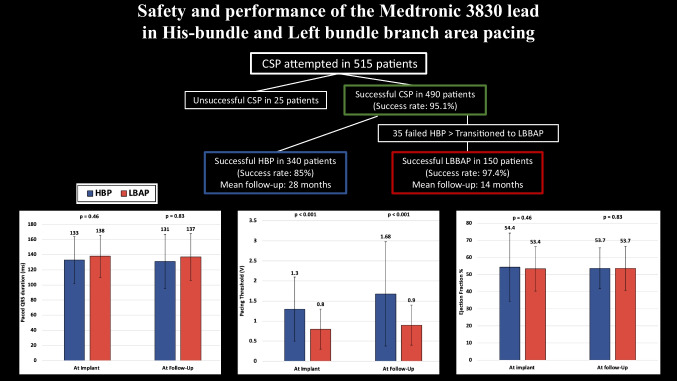

## Introduction

Conduction system pacing (CSP) represents a significant advancement in treating bradyarrhythmia. Compared to traditional right ventricular pacing (RVP), CSP has demonstrated superior outcomes, including enhanced cardiac function, reduced QRS duration, and fewer complications [1–3]. However, despite two decades of experience and growing interest, the long-term effectiveness of CSP remains underexplored due to a scarcity of large-scale, randomized controlled trials [4].

Emerging evidence from multicenter registries, long-term follow-ups, and initial RCTs has shed light on His Bundle Pacing (HBP). This technique shows promise in preventing pacing-induced cardiomyopathy and maintaining physiological inter-ventricular activation [5, 6]. Despite these benefits, HBP is constrained by challenges such as high capture thresholds, low amplitude of sensed R waves, extended use of fluoroscopy, and increased likelihood of lead revision [7, 8]. Conversely, Left Bundle Branch Area Pacing (LBAP) offers several improvements over HBP, including higher procedural success rates, improved R wave amplitudes, lower pacing thresholds, and shorter procedure times [9–12].

Advancements in pacing techniques continually seek to mitigate the well-documented adverse effects of traditional RVP [13]. The recent introduction of HBP and LBAP offers promising solutions, now reflected in updated guidelines that suggest their use in specific patient groups based on LVEF [14]. Our study adds to the emerging body of evidence, highlighting the safety and effectiveness of these innovative pacing strategies, predominantly employing the Medtronic SelectSecure 3830 lm-less lead.

## Methods

This retrospective study was conducted at Cleveland Clinic Florida–Weston, targeting all consecutive patients implanted with the Medtronic SelectSecure 3830 leads (Medtronic Inc., Minneapolis, MN) between May 2016 and October 2021. CSP was primarily utilized for patients with indications for RVP to prevent pacing-induced cardiomyopathy, with the exception of a small subset of patients who underwent AV nodal ablation and received conduction system pacing as part of a His-optimized cardiac resynchronization therapy (HOT-CRT) or Left bundle branch-optimized cardiac resynchronization therapy (LOT-CRT) systems. Our study adheres to the ethical guidelines of the 1975 Declaration of Helsinki, as reflected in the prior approval by our institution’s research board.

The primary endpoint focused on the successful implantation rate and lead performance, including sensing and capture thresholds. Safety endpoints were also crucial, covering system-related complications such as infections, lead dislodgment, increased capture threshold (> 2.5 V at 1 ms at follow-up or > 1 V from implant), premature battery depletion, and the need for lead revision. We also assessed the specific left ventricular activation time (LVAT) in the HBP and LBAP subpopulations at implantation and during follow-ups [15].

The implantation procedure involved four skilled proceduralists using a fixed curve sheath (C315 HIS, Medtronic Inc., Minneapolis, MN) for a trans-ventricular-septal approach at the ventricular septum. An intracardiac electrogram from the lead tip and a 12-lead surface electrocardiogram were concurrently recorded using a GE CardioLab EP Recording System. For HBP, the procedure aimed to identify the distal His potential, ensuring no atrial electrogram was present before securing the lead. Successful His-bundle capture, either selective or nonselective, indicated a successful procedure [16]. Successful HBP capture was defined by matching the stimulus-to-QRS duration with the His electrogram-to-QRS duration, ensuring an isoelectric interval and a paced QRS with similar morphology to the native QRS, indicative of direct His bundle engagement without myocardial involvement. Non-selective HBP was characterized by immediate onset of ventricular activation post-stimulus, with a broader QRS due to ventricular fusion, and differentiated capture thresholds for His bundle and myocardial capture. If unsuccessful, the lead was repositioned for LBAP. Initially, HBP lead output was typically set at 3.5 V at 1 ms or twice the His-bundle capture threshold, with auto-capture algorithms disabled.

LBAP involved placing the lead in the deep septal region, confirmed via fluoroscopy or echocardiography, conducted following the method outlined by Padala et al. [17], inserting the sheath into the right ventricle, advancing the pacing lead, and applying pacing at 2.0 V/0.5 ms. Successful LBAP was indicated by a specific QRS morphology in the ECG, characterized by LBBB morphology with a “W” waveform notch at the nadir of the QRS waveform in ECG lead V_1_, and by identifying a transition from nonselective to selective left bundle branch capture during threshold testing, marked by changes in QRS morphology and discrete local electrograms. In some selective cases, the efficacy of LBAP was validated by a retrograde His potential within 35 ms from the pacing artifact. Upon achieving this, the lead was secured with 4–5 clockwise rotations. A fluoroscopic radiograph confirmed the lead’s position towards the septum. Backup RV pacing leads were implanted in certain patients based on the operator’s judgment, using a CRT-P generator.

Baseline patient demographics, ECG characteristics, echocardiographic parameters, and pacing indications were documented. Additionally, lead parameters at the time of implantation were recorded; the reported threshold was the lowest voltage necessary to capture the conduction system. Post-procedure, the pacing devices were individually programmed to meet each patient’s specific needs. Patients were followed in-person in the device clinic at 1 month, 3 months, and every 6 months thereafter. During these visits, pacing parameters and 12-lead ECGs were recorded. Additionally, remote device interrogations were conducted every 3 months to ensure continuous monitoring and optimal device performance. Follow-up visits allowed for necessary adjustments, focusing on optimizing the patient’s response to CSP. This involved fine-tuning AV delay, ventricular sensitivity, and ensuring optimal outputs. The goal was to maintain an adequate safety margin of capture, thereby maximizing battery longevity.

Statistical Analysis was performed using SPSS Statistics Version 25.0 (SPSS Inc, Chicago, IL), with descriptive statistics summarizing the baseline and clinical characteristics of the patients. This included means with standard deviations for continuous variables and counts (percentages) for categorical variables. Univariable analysis was used to compare the characteristics between HBP and LBAP patients. The independent samples *t*-test or Mann–Whitney *U* test was used for continuous variables, and chi-square or Fisher’s exact tests were used for categorical variables as appropriate, depending on the distribution of the data. A *p*-value of < 0.05 was considered statistically significant.

## Results

Our study analyzed a total of 490 cases of CSP, comprising 340 in the HBP group and 150 in the LBAP group. Initially, CSP was attempted in 515 patients, but it proved unsuccessful in 25 cases. Among these unsuccessful attempts, 21 patients failed HBP implant and were subsequently transitioned to RVP. Additionally, 4 patients failed both HBP and LBAP and also moved to RVP. These 25 cases were excluded from the final analysis. Conversely, 35 patients initially unsuccessful with HBP were successfully transitioned to LBAP and were thus included in the LBAP group in our final dataset. Our cumulative data show a high success rate of CSP implantation at 95.1%, with HBP achieving an 85% success rate and LBAP demonstrating a higher success rate of 97.4% (*p* < 0.001).

Demographically, the groups did not differ significantly in age or gender distribution. Notably, the LBAP group exhibited a significantly higher incidence of congestive heart failure (CHF) (52.6% vs. 32.6% in the HBP group, *p* < 0.001) and cardiac amyloidosis (CA) (5.3% vs. 1.7% in the HBP group, *p* = 0.02). The prevalence of other comorbidities, including atrial fibrillation/flutter (AF), arterial hypertension, diabetes mellitus, thyroid disease, and chronic kidney disease, was similar across both groups. Regarding indications for CSP, sinus node dysfunction was more common in the HBP group (35.5%) than in the LBAP group (24%), (*p* = 0.11). High-degree AV block was slightly more prevalent in the LBAP group (46%) compared to the HBP group (35.2%), (*p* = 0.25). The rates of CRT and refractory AF were comparable between the groups, as was the proportion of patients with more than one pacing indication (23.5% in HBP vs. 22% in LBAP, *p* = 0.71).

Baseline electrocardiographic (ECG) characteristics such as the presence of infrahisian disease, manifested as LBBB or RBBB, and paced rhythm were consistent between the groups. However, the pre-implant QRS duration was notably longer in the LBAP group (119.5 ± 31 ms) compared to the HBP group (113 ms ± 32 ms, *p* = 0.04).

The procedural and fluoroscopy times were longer in the LBAP group, with average durations of 145 ± 43 min and 15 ± 12 min, respectively, compared to 127 ± 41 min and 11 ± 10 min in the HBP group (*p* < 0.001 for both comparisons). Single-chamber implantations were less frequent in the LBAP group (2%) compared to the HBP group (5.3%, *p* < 0.001). A higher rate of simultaneous AV node ablation was observed in the LBAP group (14% vs. 9.4% in the HBP group, *p* = 0.03). The use of backup RV leads was similar in both groups (6% in the LBAP group vs. 6.2% in the HBP group), primarily in cases presenting with high-degree AV block or undergoing simultaneous AV node ablation. The use of HOT-CRT or LOT-CRT was less common in the LBAP group (2% vs. 4.7% in the HBP group, *p* < 0.001).

The follow-up period averaged 28 ± 20 months for the HBP group and 14 ± 13 months for the LBAP group, (*p* < 0.001). The LBAP group showed a slightly higher rate of loss to follow-up (8% vs. 5.3% in the HBP group, *p* < 0.001). LVEF remained stable and comparable between the groups from implantation to follow-up (53.4 ± 12.9% in the LBAP group vs. 54.4 ± 12% in the HBP group initially, *p* = 0.46; 53.7 ± 12.9% in the LBAP group vs. 53.7 ± 11.4% in the HBP group at follow-up, *p* = 0.83).

Within the first 30 days post-implant, two HBP patients developed pneumothorax, which was successfully managed non-surgically. One HBP patient experienced a pocket hematoma that was effectively treated with oral antibiotics. There were no early reports of device-related infections in either group. Lead dislodgment within the first month occurred in three cases: two in the HBP group (one re-implanted as HBP, one as LBAP) and one in the LBAP group, who underwent re-implantation as LBAP. Each group reported one death within 30 days of implantation; both for causes not related to the implant procedure or the device/lead performance.

From 2 months to 3 years post-implant, lead revisions were necessary in 50 HBP cases and 5 LBAP cases, primarily due to increased capture thresholds, loss of capture, and exit block. Exit block at follow-up was notably higher in 40 HBP patients (11.7%) compared to 5 LBAP patients (3%), (*p* < 0.001). Early replacement indicator (ERI) events occurred in 5 cases of HBP (1.5%); and no ERI events reported in the LBAP group within the first 5 years. Lead extractions were successfully performed in 3 HBP cases, with no extraction attempts in the LBAP group.

Table 1 presents a summary of baseline participant characteristics, prevalence of comorbidities, procedural details, and complications.

**Table 1:** Baseline participants’ characteristics, procedure’s details and complications within 30-days of implant

Variables	HBP (*n* = 340)	LBBAP (*n* = 150)	*p*-value
**Baseline characteristics**			
**Demographics**			
Age at implant	75 ± 9.9	77 ± 10	0.03
Male gender	206 (60.5%)	94 (62.6%)	0.66
**Comorbidities**			
Hypertension	233 (68.5%)	112 (74.6%)	0.17
Diabetes	89 (26.1%)	48 (32%)	0.18
Congestive heart failure	111 (32.6%)	79 (52.6%)	< 0.001
LVEF at implant	54.4 ± 12	53.4 ± 12.9	0.46
AFib/flutter	206 (60.5%)	103 (68.6%)	0.108
Cardiac amyloidosis	6 (1.7%)	8 (5.3%)	0.02
Thyroid disease	84 (24.7%)	43 (28.6%)	0.35
Creatinine	1.17 ± 0.82	1.17 ± 0.54	0.92
**Indications**			
Sinus node dysfunction	121 (35.5%)	36 (24%)	0.11
High degree AV block	120 (35.2%)	69 (46%)	0.25
Cardiac resyncronization therapy	34 (10%)	15 (10%)	1
Refractory AF	64 (18.8%)	30 (20%)	0.76
> 1 indication	80 (23.5%)	33 (22%)	0.71
**Baseline electrocardiogram**			
Pre-implant QRS duration (ms)	113.1 ± 32.6	119.5 ± 31.7	0.04
Left bundle branch block	41 (12.05%)	26 (17.33%)	0.11
Right bundle branch block	61 (17.9%)	33 (22%)	0.3
Paced	17 (5%)	10 (6.6%)	0.47
**Procedure details**			
Procedure time	127 ± 41	145 ± 43	< 0.001
Fluoroscopy time	11.3 ± 9.9	15 ± 12.4	< 0.001
Simultaneous AV node ablation	32 (9.4%)	21 (14%)	0.03
Single-chamber	18 (5.3%)	3 (2%)	0.09
Back-up RV lead	21 (6.2%)	9 (6%)	0.94
HOT-CRT or LOT-CRT	16 (4.7%)	3 (2%)	< 0.001
**Complications**			
**Within 30 days of implant**			
Pneumothorax	2 (0.6%)	0%	0.34
Pocket hematoma	1 (0.3%)	0%	0.5
Device related infections	0%	0%	1
Death < 30 days	1 (0.3%)	1 (0.66%)	0.55
**Reasons for lead revision**			
Exit block	40 (11.7%)	5 (3%)	< 0.001
Lead extraction	5 (1.5%)	0	< 0.001
Early ERI (< 5 years)	5 (1.5%)	0	< 0.001

**Fig. 1:**
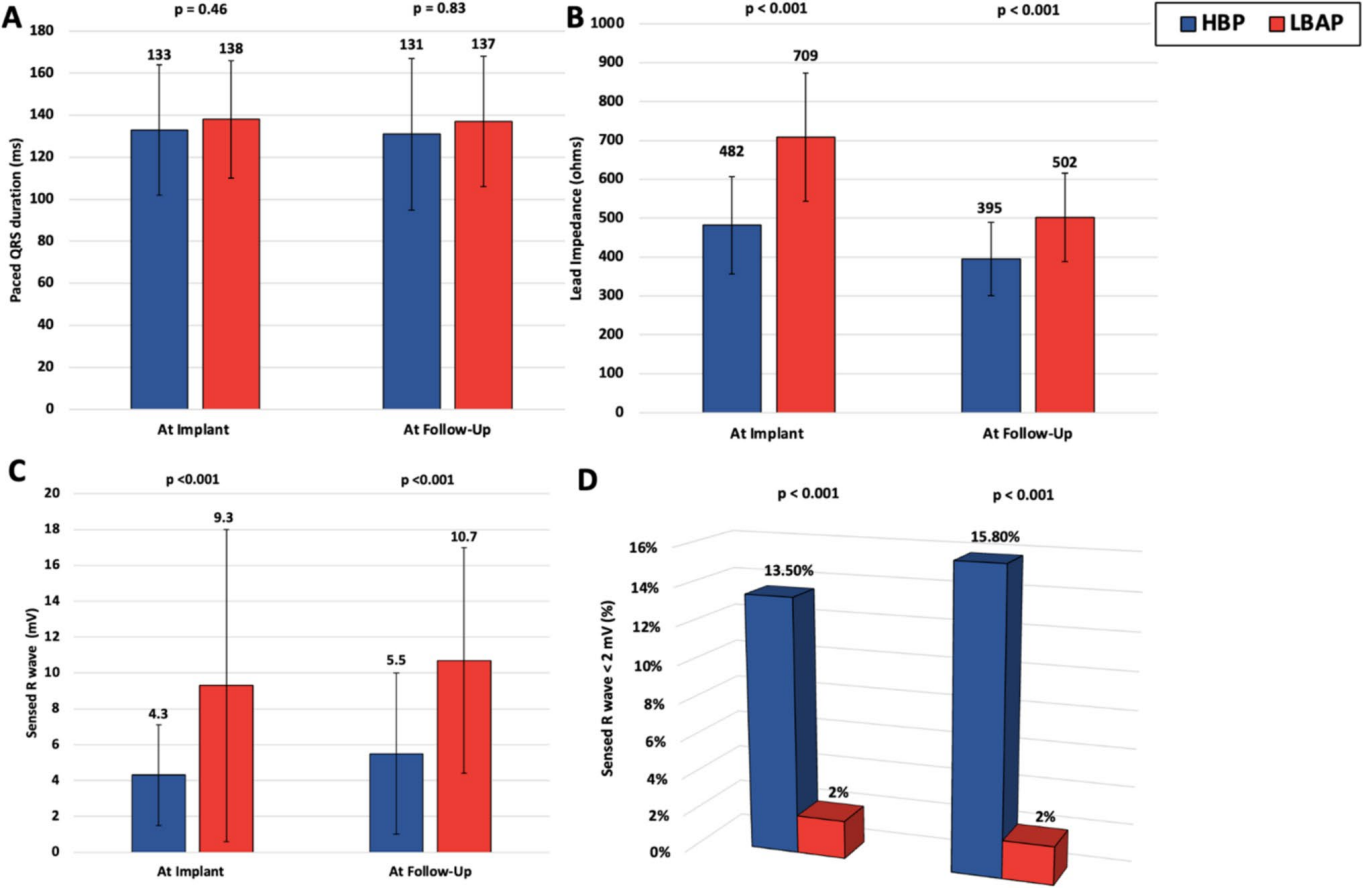
Comparative analysis of pacing parameters during implantation and follow-up. A Stable QRS durations in both HBP and LBAP from implantation to follow-up. **B** Consistent lead impedance levels for both pacing methods. **C** Lower sensed R-wave amplitudes in HBP than LBAP, indicating sensing challenges in HBP. **D** A higher proportion of HBP patients with R-wave amplitudes below 2 mV

The post-implant and follow-up paced QRS durations were similar between groups. In the HBP group, there was a minor reduction in QRS duration from 133 ± 31 ms at implantation to 131 ± 36 ms at follow-up. With LBAP the QRS duration decreased from 138 ± 28 ms to 137 ± 31 ms. Both groups experienced a decrease in impedance, from 482 ± 125 Ω to 395 ± 94 Ω in the HBP group and from 709 ± 165 Ω to 502 ± 114 Ω in the LBAP group. Initial R-wave amplitudes in the HBP group were 4.3 ± 2.8 mV, increasing to 5.5 ± 4.5 mV at follow-up (*p* = 0.09), with 13.5% of patients initially and 15.8% at follow-up having R-wave amplitudes below 2 mV. The LBAP group started with higher R-wave amplitudes (9.3 ± 8.7 mV), increasing to 10.7 ± 6.3 mV during follow-up (*p* = 0.007), with no change in the incidence of R-wave amplitudes below 2 mV. Figure 1 displays these trends.

Pacing thresholds in the HBP group increased from 1.3 ± 0.8 V at 0.8 ± 0.2 ms to 1.68 ± 1.3 V at 0.7 ± 0.3 ms. Conversely, the LBAP group maintained stable thresholds (0.8 ± 0.5 V at 0.5 ± 0.3 ms initially and 0.9 ± 0.5 V at 0.5 ± 0.3 ms at follow-up), leading to a significant difference in the proportion of patients with thresholds > 2.4 V at the latest follow-up (18.8% in the HBP group vs 2.6% in the LBAP group, p < 0.001). Table 2 andFig. 2 provide detailed comparisons of pacing thresholds and other lead parameters between the groups.

**Table 2:** Follow-up and lead performance

Long-term follow-up and lead performance			
**Variables**	**HBP (*n* = 340)**	**LBBAP (*n* = 150)**	***p*-value**
Follow up (months)	28 ± 20	14 ± 13	< 0.001
Loss to follow-up	18 (5.3%)	12 (8%)	< 0.001
Pacemaker dependent	181 (50%)	94 (62.6%)	0.03
**Parameters at implant**			
Post-implant paced QRS (ms)	133 ± 31	138 ± 28	0.09
Sensed R wave (mV)	4.3 ± 2.8	9.3 ± 8.7	< 0.001
Sensed R wave < 2 mV	46 (13.5%)	3 (2%)	< 0.001
Impedance (ohms)	482 ± 125	709 ± 165	< 0.001
Pacing threshold (V)	1.3 ± 0.8	0.8 ± 0.5	< 0.001
Pulse duration(ms)	0.8 ± 0.2	0.5 ± 0.3	< 0.001
**Parameters at the latest follow-up**			
LVEF at follow-Up	53.7 ± 11.4	53.7 ± 12.9	0.83
Follow-up paced QRS (ms)	131 ± 36	137 ± 31	0.118
Sensed R wave (mV)	5.5 ± 4.5	10.7 ± 6.3	< 0.001
Sensed R wave < 2 mV	54 (15.8%)	3 (2%)	< 0.001
Impedance (ohms)	395 ± 94	502 ± 114	< 0.001
Pacing threshold (V)	1.68 ± 1.3	0.9 ± 0.5	< 0.001
Pulse duration (ms)	0.7 ± 0.3	0.5 ± 0.3	< 0.001
Pacing threshold > 2.4 V	64 (18.8%)	4 (2.6%)	< 0.001

**Fig. 2:**
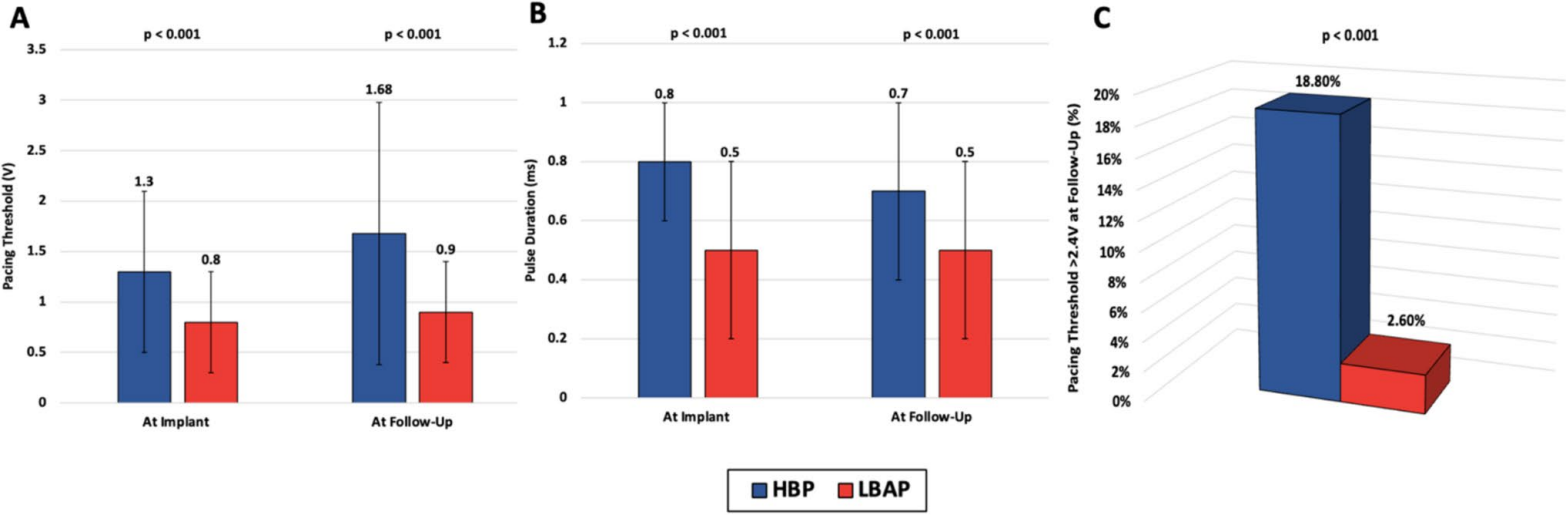
Detailed analysis of pacing threshold variations over time. **A** LBAP had lower pacing thresholds than HBP, with HBP experiencing an increase at follow-up. **B** Unchanged pulse durations from implantation to follow-up in both groups. **C** More HBP patients had thresholds above 2.4 V at follow-up

In a subgroup analysis, 191 patients from the HBP group and 98 patients from the LBBAP group had over 80% ventricular pacing burden. The LBAP group showed a slightly longer pre-implant QRS duration (125 ± 33 ms) compared to the HBP group (118 ± 33 ms, *p* = 0.08). Post-implant paced QRS durations were longer with LBAP (145 ± 22 ms) compared to HBP (140 ± 29 ms, *p* = 0.38). Moreover, a higher proportion of HBP patients had a R-wave peak time in lead V_6_ < 80 ms after implantation (66% vs. 47% in the LBAP group, *p* = 0.02).

## Discussion

Our retrospective single-center study examines the intermediate-term performance and safety of HBP and LBAP using Medtronic’s SelectSecure 3830 lumenless leads in a substantial cohort of 490 patients. It is important to note that the criteria for LBAP lead position have evolved since our initial implantations in 2020, encompassing selective and non-selective LBB and left septal capture, though clinical outcomes data are not claimed. In mid-2018, we began transitioning to LBAP for patients where HBP was not feasible, initially adopting it as deep septal pacing. By mid-2020, LBAP had become our primary pacing approach.

Over an average follow-up of 28 months for the HBP group and 14 months for the LBAP group, our observations revealed significant findings: HBP capture thresholds increased notably, with 18.8% of patients exceeding a 2.5 V threshold; LBAP demonstrated consistently lower pacing thresholds and higher R-wave amplitudes; and while both techniques were safe, HBP had a lower implantation success rate and a higher incidence of lead revision compared to LBAP.

Existing literature primarily offers observational data with a lack of long-term lead survival information [14]. However, our findings suggest that both HBP and LBAP provide physiological pacing that could mitigate the adverse effects typically associated with RVP. Moreover, LBAP may present additional benefits such as lower thresholds and greater stability, potentially making it a more favorable option than HBP [18].

Our study achieved implantation success rates of 85% for HBP and 97.4% for LBAP. These results show that while the success rate for LBAP aligns with other institutions [19, 20], our success rate for HBP notably exceeds the typical 50% reported elsewhere [21]. We attribute this higher success rate to the extensive experience and persistence of our implantation team, who were willing to make multiple attempts and spend the necessary time to ensure success. The lower success rates and higher thresholds observed in HBP are linked to anatomical challenges, including the small and variably positioned His bundle [22], which is often encased in dense, electrically insulating fibrous tissue [23].

Interestingly, the procedural and fluoroscopy times were initially shorter for HBP compared to LBAP. This disparity is largely due to the absence of definitive electrocardiographic criteria for LBB capture and a steeper learning curve associated with LBAP. However, as our team’s experience with LBAP has grown, procedural times have decreased, highlighting the impact of learning and adaptation on procedural efficiency. To mitigate the risk of lead dislodgement, especially during the initial phase of our experience, we placed a backup RV lead in patients undergoing AV nodal ablation or those presenting with complete AV block. This strategy has proven safe, with no critical events occurring among the 30 patients who received a backup RV lead, 21 with HBP and 9 with LBAP, and no cases of lead dislodgement were observed.

In our study, LBAP was characterized by lower and more consistent pacing thresholds, in terms of both voltage and pulse width, compared to HBP. Specifically, the average pacing threshold for HBP was 1.3 V at implantation, which significantly increased to 1.68 V over a median follow-up of 28 months, with 18.8% of patients experiencing capture thresholds exceeding 2.5 V. This finding aligns with those from Padala et al. [8] and is corroborated by other studies [10]. In contrast, the LBAP group maintained stable pacing thresholds, with an initial average of 0.8 V, slightly rising to 0.9 V over a 14-month median follow-up, with only 2% of patients exceeding a 2.5 V threshold. Supporting evidence from multicenter studies, including those by Padala et al. [19] and Molina-Lerma et al. [24], confirm the safety and efficacy of LBAP, noting its superior pacing performance. Our findings are in line with their research and further emphasize LBAP’s consistently lower pacing thresholds. Additional studies by Zanon et al. [5] and Hua et al. [25] suggest LBAP as a viable alternative to both HBP and biventricular pacing, a sentiment echoed by Su et al. [26], who recognize LBAP’s safety and feasibility over prolonged follow-up periods. Our results affirm these observations, reinforcing LBAP’s potential advantages over traditional methods. Also of relevance, the post-implant QRS duration for duration for LBAP showed an average increase from 119 to 138 ms, measured using calipers on 12-lead ECGs. This increase of 19 ms, while noticeable, falls within expected variations for pacing-induced cardiomyopathy prevention, typical among early adopters of CSP techniques. We believe this is a reflection of non-selective capture and the learning curve associated with adopting new pacing methodologies and highlights the importance of documenting procedural evolution in CSP.

In our study, complications were rare and generally minor across HBP and LBAP groups, with no significant differences in the incidence of pneumothorax, pocket hematoma, or device-related infections. Both groups experienced a single death within 30 days post-implantation, neither related to the procedure or device function. The lower frequency of device revisions and extended device longevity with LBAP suggest it might be a more cost-effective option, potentially offering economic benefits.

The observed higher lead impedances and lower pacing thresholds in the LBAP group suggest a better lead-myocardial interface with reduced current drain, potentially extending battery life [27]. However, it is notable that only 5 out of the 340 participants with HBP required early battery replacement, indicating the efficacy of HBP despite its longer follow-up period (28 months compared to 14 months for LBAP). So, the role of HBP should not be hastily disregarded, also taking into account that it may be a physiologically superior pacing modality [23]; supporting this, our subgroup analysis of patients requiring more than 80% ventricular pacing found that 66% of HBP cases had an R-wave peak time in Lead V_6_ of less than 80 ms at implantation, suggesting better electrical synchronization than LBAP. In our opinion, while a randomized controlled trial comparing HBP directly may never occur, our evidence supports HBP use, particularly in patients with atrioventricular intranodal disease, where its physiological benefits are most pronounced.

Regarding cardiac function, LVEF remained stable and comparable between HBP and LBAP groups throughout the study period. As of 2023, only LBAP has received a Class 2b recommendation as an alternative to RVP for patients with normal LVEF who require minimal ventricular pacing. However, both HBP and LBAP are recommended for patients with LVEF between 36 and 50% who anticipate limited ventricular pacing. In our view, more definitive studies are needed to properly adjudicate lead conduction system capture, especially since deep septal capture may suffice for patients with preserved LVEF looking to avoid the adverse effects of RVP. Meanwhile, direct LBB capture, whether selective or non-selective, may be necessary for patients requiring cardiac resynchronization to improve reduced LVEF.

The interpretability of our findings is constrained by the observational nature of our study. While the sample size is substantial, it may still be insufficient to detect subtle differences between groups. The novelty of this technology and its learning curve may also explain some observed disparities. Our results should not be generalized across different leads or other manufacturers’ CSP technologies. Our single-center data may not be representative of other populations. It is also crucial to consider that some patients were lost to follow-up, potentially introducing bias into the results.

## Conclusion

Our study confirms that HBP and LBAP using Medtronic SelectSecure 3830 leads are safe, feasible, and reproducible. Drawing from a substantial patient cohort, we found that LBAP, in particular, may offer advantages over HBP, including a more stable lead-myocardial interface, fewer device revisions, and extended device longevity. These benefits align with observations from other research groups. However, further investigations are needed to refine lead position adjudication and to verify conduction system capture in LBAP.

## Data Availability

Data will be available upon reasonable request to the corresponding author.

## Abbreviations

CHF: Congestive heart failure
CSP: Conduction system pacing
HBP: His bundle pacing
LBAP: Left bundle branch area pacing
LVAT: Left ventricular activation time
RVP: Right ventricular pacing
